# Modeling Threats to AI-ML Systems Using STRIDE †

**DOI:** 10.3390/s22176662

**Published:** 2022-09-03

**Authors:** Lara Mauri, Ernesto Damiani

**Affiliations:** 1 Department of Computer Science, Università Degli Studi di Milano, 20133 Milan, Italy; 2 Center for Cyber-Physical Systems, Khalifa University of Science and Technology, Abu Dhabi 127788, United Arab Emirates

**Keywords:** artificial intelligence security, threat modeling, vulnerability assessment

## Abstract

The application of emerging technologies, such as Artificial Intelligence (AI), entails risks that need to be addressed to ensure secure and trustworthy socio-technical infrastructures. Machine Learning (ML), the most developed subfield of AI, allows for improved decision-making processes. However, ML models exhibit specific vulnerabilities that conventional IT systems are not subject to. As systems incorporating ML components become increasingly pervasive, the need to provide security practitioners with threat modeling tailored to the specific AI-ML pipeline is of paramount importance. Currently, there exist no well-established approach accounting for the entire ML life-cycle in the identification and analysis of threats targeting ML techniques. In this paper, we propose an *asset-centered* methodology—*STRIDE-AI*—for assessing the security of AI-ML-based systems. We discuss how to apply the FMEA process to identify how assets generated and used at different stages of the ML life-cycle may fail. By adapting Microsoft’s STRIDE approach to the AI-ML domain, we map potential ML failure modes to threats and security properties these threats may endanger. The proposed methodology can assist ML practitioners in choosing the most effective security controls to protect ML assets. We illustrate STRIDE-AI with the help of a real-world use case selected from the TOREADOR H2020 project.

## 1. Introduction

Machine Learning (ML) plays a major role in a wide range of application domains. However, when ML models are deployed in production, their assets can be attacked in ways that are very different from asset attacks in conventional software systems. One example is training data sets, which can be manipulated by attackers well before model deployment time. This attack vector does not exist in conventional software, as the latter does not leverage training data to learn. Indeed, the attack surface of ML systems is the sum of specific attack vectors where an unauthorized user (the “attacker”) can try to inject spurious data into the training process or extract information from a trained model. A substantial part of this attack surface might lie beyond the reach of the organization using the ML system. For example, training data or pre-trained models are routinely acquired from third parties and can be manipulated along the supply chain. Certain ML models rely on sensor input from the physical world, which also makes them vulnerable to manipulation of physical objects. A facial recognition camera can be fooled by people wearing specially crafted glasses or clothes to escape detection. The diffusion of machine learning systems is not only creating more vulnerabilities that are harder to control but can also—if attacked successfully—trigger chain reactions affecting many other systems due to the inherent speed and automation.The call to improve the security of Artificial Intelligence (AI) systems [1] has attracted widespread attention from the ML community, which has given rise to a new vibrant line of research in security and privacy of ML models and related applications. The ML literature is now plentiful with research papers addressing the security of specific ML models, including valuable survey papers discussing individual vulnerabilities and possible defensive techniques. However, existing work mainly focuses on ingenious mechanisms that allow attackers to compromise the ML-based systems at the two core phases of the learning process, that is the training and the inference stages. In this work, we focus on the distinct, though related, problem of defining a practical methodology for assessing ML-based systems’ security. We approach the problem from the point of view of the security practitioner who has to deal with ML-based systems rather than from the one of the AI expert dealing with security. We argue that, given the current variety and scope of threats and attacks to ML models, there is some confusion about what exactly the security analyst is expected to do to alleviate them. The goal of this paper is to describe an *asset-centered* methodology for identifying threats to ML-based systems. Our proposal is based on STRIDE [2], a well-known and widely used approach to threat modeling originally developed by Microsoft. STRIDE has been identified by independent agencies like the European Union Agency for Cybersecurity (ENISA) as a promising starting point for AI threat modeling. We argue that our extension to the original STRIDE provides an *ML-specific*, *security property-driven* approach to threat detection which can also provide guidance in selecting the security controls needed to alleviate the identified threats. This paper is an extended version of the paper entitled “STRIDE-AI: An Approach to Identifying Vulnerabilities of Machine Learning Assets” presented at the 2021 IEEE International Conference on Cyber Security and Resilience (CSR) [3].

The paper is organized as follows. In Section 2, we review existing work in the field of adversarial machine learning and ongoing research on threat modeling AI/ML systems. Section 3 describes the different stages of our reference ML life-cycle architecture and the relevant ML assets generated therein. In Section 4, we illustrate how to apply the Failure Mode and Effects Analysis approach to AI-ML systems, providing facilitation questions and sample answers along with failure modes specific to ML data, model, and artefact assets. Section 5 describes the methodology (STRIDE-AI) we propose to identify threats to ML assets. Section 6 applies STRIDE-AI to a real-world use case selected from the AI-ML applications developed in the TOREADOR H2020 project, with a focus on data and model assets. Finally, Section 7 presents comments and discussions on the proposed security methodology, while Section 8 concludes our work and proposes future research directions motivated by our efforts toward advancing the development of secure ML-based systems.

## 2. Related Work

Supervised machine learning models are known to be vulnerable to attacks based on training examples crafted or manipulated by attackers. This type of *adversarial exploitation* is well documented for various applications. These include antivirus engines, autonomous bots, visual recognition and social networks, among others [4,5,6,7,8]. These attacks have motivated research on ML security [9,10,11,12], establishing the novel research field of *Adversarial Machine Learning* (AML), which lies at the intersection of machine learning and computer security. An overview of the evolution of active research in this area over the last ten years can be found in [13], where the authors presented a historical picture of the work related to the security of machine learning from a technical perspective. To summarize their findings, current ML security research aims to address the following issues: *(i)* identifying potential weaknesses of ML-based systems, *(ii)* devising the corresponding adversarial attacks and evaluating their impact on the attacked system, and *(iii)* proposing countermeasures against the considered attacks. The issue of adversarial attacks has recently attracted a considerable interest, resulting in the publication of a number of papers proposing novel types of attack and defense mechanisms for specific ML algorithms [14,15,16,17,18]. This line of work, which mainly focuses on identifying data-, model-, and system- oriented attacks and defenses [19,20,21], represents one of the two core branches into which research in the field of ML security can be divided. Researchers in this first area typically address the security concerns with a narrow focus on the specific type of compromise to be achieved or addressed, neglecting a thorough investigation of secure development practices that incorporate identification of both ML-specific and traditional system threats [22,23].

Another emerging research line focuses on threat analysis techniques that can support security expert’s understanding of how ML-based systems may fail. Some recent work explore the applicability of threat modeling methodologies traditionally used in the software engineering area to ML-based systems’ security. These works associate threats to artefacts produced at the different stages of ML models’ life-cycle, from requirements analysis to system maintenance (see Section 1). Wilhjelm et al. [24] apply a traditional threat modelling method together with the corresponding attack libraries to understand the security of ML-based systems at the requirements phase. In order to rank the impact of the identified threats, the authors make use of a *bug bar* [25] for associating severity levels to threats. Our own recent work [26] focuses instead on the ML models’ maintenance stage, proposing a metric based on the notion of a “gold standard” data set for assessing ML models’ degradation in production. Bitton et al. [27] perform a systematic ML-oriented threat analysis for the Open Radio Access Network (O-RAN) architecture, identifying potential threat actors in the O-RAN ecosystem and mapping them to the capabilities needed by the attacker. The study by Chen and Ali Babar [28] presents a holistic view of system security for ML-based modern software systems. The architectural risk analysis for ML security [29], proposed by the Berryville Institute of Machine Learning, is designed for use by developers, engineers, and others creating applications and services that rely on ML models. Specifically, the Berryville analysis lists 78 risks identified using a generic ML system as an organizing concept, and then identifies the top ten.

The well-known MITRE Adversarial Tactics, Techniques, and Common Knowledge (ATT&CK) framework [30] was created by MITRE to document adversary tactics and techniques based on real-world observations. The MITRE framework provides a common taxonomy of adversarial actions understood by both offensive and defensive sides of cybersecurity, and can be used as a tool to analyze adversary behavior. Complementary to the MITRE ATT&CK framework is MITRE ATLAS (Adversarial Threat Landscape for Artificial-Intelligence Systems) [31], a knowledge base of adversary tactics and techniques which includes demonstrations from red teams and security groups, real-world observations and the state of academic research findings. ATLAS includes some AI/ML case studies. In the threat landscape report on AI, published in late 2020 [32], the European Union Agency for Network and Information System security (ENISA) sets a baseline for a common understanding of security threats to AI, identifying a list of AI-ML assets in the context of all stages of the ML models life-cycle and mapping threats against them. Another recent ENISA’s report [33] presents a detailed analysis of threats targeting ML-based systems (including data poisoning, adversarial attacks and parameters’ exfiltration) and provides a list of security controls described in the literature. Further efforts to understand how ML-based systems may fail and, consequently, how to respond to such failures have been made by Microsoft through the release of guidelines for the mitigation and triage of AI-specific security threats [34]. Microsoft’s approach, which is developed, like ours, on STRIDE threat-modeling [2], does not address the security properties definition. Rather, it is based on a taxonomy that classifies ML failure modes into two categories, namely *intentional* and *unintentional* failures. Intentional failures are mapped to a list of attacks reported in the literature [35].

## 3. ML-Based Application Life-Cycle and Related At-Risk Assets

**Figure 1:**
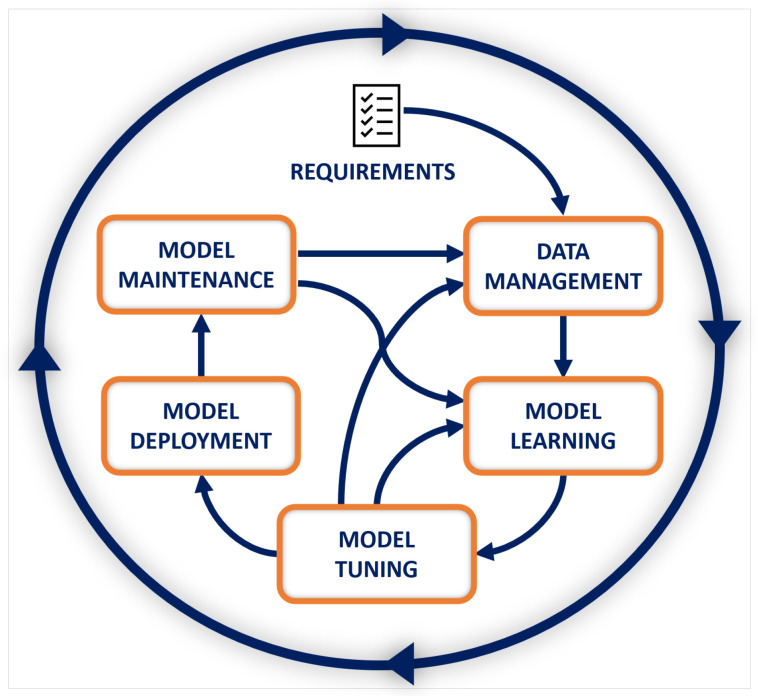
Our reference ML life-cycle [3].

Although there exist many diverse types of learning tasks [36], the development process of ML-based systems has an intrinsic iterative multi-stage nature [37]. Figure 1 shows our reference *ML life-cycle*, starting from requirements analysis and ending with the ML model’s maintenance in response to changes. While this life-cycle does not cover all possible developments, we will use it to identify the key data assets produced at each phase and to analyze their failure modes. We start by outlining the activities carried out at each stage.

The initial stage of the ML life-cycle, *Data Management*, includes a number of steps, a major one being the *ingestion* of the data required for the next stages. Ingestion occurs from multiple sources, and the data collected can either be stored or immediately used. Pre-processing techniques are used to create a consistent data set suitable for training, testing and evaluation. The next step, *Model Learning*, involves developing or selecting an ML model that can handle the task of interest. Depending on the goals and the amount and nature of the knowledge available to the model, different ML techniques can be used, such as supervised, unsupervised and reinforcement learning. In the training process of a supervised ML-based system, a learning algorithm is provided with predefined inputs and known outputs. The learning algorithm computes some *error metrics* to determine whether the model is learning well, i.e., it delivers the expected output not only on the inputs it has seen in training, but also on test data it has never seen before. The so-called *hyper-parameters*, which control how the training is done (e.g., how the error is used to modify the ML model’s internal parameters), are fine-tuned during the *Model Tuning* stage. While being tuned, the ML model is also validated to determine whether it works properly on inputs collected independently from the original training and test sets. The transition from development to production is handled in the *Model Deployment* stage. In this stage, the model executes *inferences* on real inputs, generating the corresponding results. As the production data landscape may change over time, in-production ML models require continuous monitoring. The final ML life-cycle stage, *Model Maintenance*, monitors the ML model and retrains it when needed.

A number of attack surface and attack vectors can be identified along a typical ML life-cycle. On one hand, some of the potential vulnerabilities are already known to exist in conventional IT systems and still remain part of the ML attack surface, though perhaps they can be seen in a new light when examined through the ML lens. On the other hand, this traditional attack surface expands along new axes when considering the specific, multifaceted and dynamic nature of ML processes. The resulting surface is therefore extremely complex, and mapping it requires going through all the various steps of the ML life-cycle and explaining the different security threats, a task that is inherently challenging due to the large amount of vectors that an adversary can target. Regardless of the ML stage targeted by the adversary, attacks against ML-based systems have negative impacts that generally result in performance decrease, system misbehavior, and/or privacy breach.

**Figure 2:**
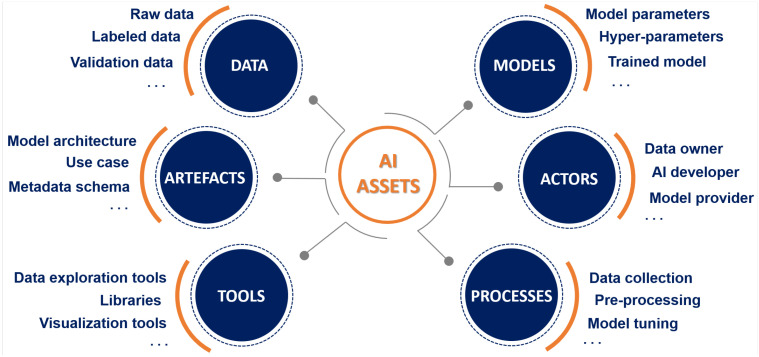
Assets in the AI ecosystem [3].

At each stage of the ML life-cycle, multiple *digital assets* are generated and used. Identifying assets in the context of the diverse ML life-cycle stages (including inter-dependencies between them) is a key step in pinpointing what needs to be protected and what could go wrong in terms of security of the AI ecosystem. Based on the generic ML life-cycle reference model described above (see Figure 1), at-risk ML assets can be grouped into six different macro-categories—*Data*, *Models*, *Actors*, *Processes*, *Tools*, and *Artefacts*—as shown in Figure 2. It should be noted that, given the complex and evolving nature of ML-based systems, proper identification of asset that are subject to ML-specific threats must be considered an ongoing task that needs to keep pace with developments in AI/ML solutions.

## 4. Failure Mode and Effects Analysis of AI-ML Systems

Failure Mode and Effects Analysis (FMEA) is a well-established, structured approach to discovering potential failures that may exist in the design of a product or process [38,39]. *Failure modes* are the ways in which an asset (be it a process, system or component) can fail. *Effects* are the ways that these failures can lead to waste or harmful outcomes for the customer. FMEA techniques are intended to identify, prioritize and limit failure modes of manufacturing or engineering processes, products, designs or services in a systematic way by determining their potential occurrence, root causes, implications and impact [40]. In order to establish the next actions to be made, a quantitative score is calculated to evaluate failures on the basis of their severity.

### 4.1. Guide to FMEA Application in the AI-ML Life-Cycle

FMEA first emerged in the military domain and then spread to the aerospace industry and to other manufacturing domain, with various applications in the nuclear electronics, and automotive fields as well. Recently, researchers have explored how FMEA or other safety engineering tools can be used to assess the design of AI-ML systems [41,42]. Applying FMEA to an AI-ML asset includes the following activities: *(i)* assigning functions to the asset, *(ii)* creating structure, function, networks diagrams for the asset, *(iii)* define defects that can cause the asset’s function/function network to fail, *(iv)* perform threat modeling actions. Specifically, the above operations can be accomplished by performing the following steps:

- Step 1: Create one function list per asset. The content of these function lists should be different for each asset in at least a function;
- Step 2: Specify prerequisites for functions that may refer to functions of other assets. This is the basis used to create function networks;
- Step 3: Identify one or more asset defects that can impair a function (Failure Mode—FM). Add one or more causes or effects for each defect;
- Step 4: Use a threat-modeling methodology to map FMs to threats.

The steps of FMEA involve different roles, including the asset category owners (Step 1), the action managers (Steps 2 and 3) and the security analysts (Step 4). A *severity score* can be assigned to FMs based on the failure effects. This assignment is done independently from the severity assessment of the threats (see Section 6.3). However, to ensure that the two evaluations are consistent, FM severity should be passed on from the FMs to the threats associated to them, e.g., as a lower bound to DREAD-estimated threat severity.

### 4.2. Question Facilitator

In order to support practitioners in performing an effective FMEA [43] analysis, we identified some key questions to ask as part of the FMEA procedure.   

**Functions.** When identifying functions for System or Design FMEAs, refer to the AI-ML life-cycle and to the asset list of your application

- *What is the primary purpose of this asset?*
- *What is the asset supposed to do? What must the asset not do?*
- *What functions occur at the interfaces?*
- *What is the standard of performance?*

**Failure Modes.** When identifying FMs refer to the architecture diagram of your AI-ML application

- *In what way could the asset fail to perform its intended function?*
- *In what way could the asset perform an unintended function?*
- *What could go wrong with this asset?*
- *What could go wrong at the asset’s interfaces?*
- *What has gone wrong with this asset in the past?*
- *How could the asset be abused or misused?*
- *What concerns do you have with this asset?*

**Effects**. When identifying effects for FMs, refer to the FM list identified in the previous step

- *What is the consequence of the failure?*
- *If the asset fails, what will be the consequences at the local level? At the next higher level? At the overall system level? At the end user?*
- *If the asset fails, what will the customer see, feel or experience?*
- *Will the asset failure cause potential harm to the end users?*
- *Will the asset failure cause potential violation of regulations?*

### 4.3. ML Assets and Their Failure Modes

Following the FMEA procedure, once assets have been identified and their intended role within the ML life-cycle is fully understood, failure mode analysis involves pinpointing defects and errors, potential or actual, that may exist. For illustrative purposes, we select three of the identified ML asset categories shown in Figure 2, namely data, models and artefacts—of which certainly the former is the most imperiled asset, as its compromise can lead to potentially catastrophic consequences for the trained model. For each of the at-risk assets comprised therein we show how the use of failure conditions may aid in identifying their relevant failure modes. To be precise, here we care about illustrating the procedure that guides ML practitioners to identify possible failure modes (useful for subsequent threat analysis), temporarily neglecting the study of the consequences of the identified FMs. Specifically, in Table 1, Table 2 and Table 3 we provide the asset description, answers to the corresponding FMEA asset questions (see Section 4.2), as well as a set of possible FMs for the data, model, and artefact assets, respectively. Of course, the manner in which the asset does not accomplish its intended function, that is, how the specific asset may fail to hold the properties needed for the correct execution of a stage of the ML model’s life-cycle, is impacted by the nature of the cause of the failure itself. As shown in the third column of each of Table 1, Table 2 and Table 3, FMs can result from the innate design of the system or by the presence of adversarial intention. Thus, FMs may include improper or poor performance of functions as well as execution of unintended or undesired functions that ultimately also reflect assumptions made about the attacker’s capabilities and strategies.

## 5. Modeling Threats to ML Assets

We now discuss how the ML assets’ failure modes can be used to identify threats. Classically, *Threat Modeling* (TM) is the process of reviewing the security of a system, identifying critical areas, and assessing the risk associated with them. TM is a fundamental phase in the design of information systems because it allows profiling and prioritizing problems as well as assessing the value of potential mitigation in alleviating threats. A typical TM process [44] consisting of five steps is shown in Table 4.

Several TM methods are available. Popular approaches include PASTA [45], a risk-centered TM framework consisting of seven stages within which different elicitation tools are used, and OCTAVE [46], which is a three-phase method focusing on the assessment of the organizational risks rather than the technological ones. Originally defined by Loren Kohnfelder and Praerit Garg [47,48], STRIDE is the most mature one. It has been applied to many vertical domains, including cyber-physical systems and healthcare applications [49,50,51,52,53]. STRIDE uses a set of six threats based on its acronym, which stands for *Spoofing*, *Tampering*, *Repudiation*, *Information Disclosure*, *Denial of service*, and *Elevation of privilege*; Table 5 shows their definitions. Our discussion of the ML life-cycle and its key assets (Section 3) has covered steps 2 and 3 of the TM process. We need to set the security objectives (step 1), and then proceed with the threat identification step (step 4) and the vulnerability identification one (step 5). We start by proposing our ML-specific definitions of the classic $CIA^{3}-R$ security properties (step 1). Then, we will discuss how assets’ failure modes can jeopardize these properties. Finally, we will use the results of our analysis for identifying threats to the ML life-cycle (step 4).

### 5.1. Extending STRIDE to ML-Based Systems

The $CIA^{3}-R$ hexagon [54] includes the six main components of security. Each STRIDE threat corresponds to the violation of a $CIA^{3}-R$ security property, as shown in Table 6.

While STRIDE can be directly applied to vertical domains, applying it to AI-ML requires customization of the desired set of properties in order to match the domain assets’ specific failure modes [55]. Below, we propose our ML-specific definition of $CIA^{3}-R$ security properties. Then, we map the failure modes of the assets under consideration—data, model, artefact—generated in our ML life-cycle (Section 3) to the violation of one or more of these properties. For each mapping, we associate (the effects of) the corresponding failure to one or more STRIDE threats to ML-based systems. Table 7 shows our proposed definition of an ML-specific $CIA^{3}-R$ hexagon.

For clarity, in the context of authenticity, we assume the ML model output is verifiable in the sense that results come with certain genuineness and have the property of being able to be trusted. This implies a degree of confidence in the validity of predictions, though this may be affected when integrity is compromised. As for availability, our definition is tied to the usage scenarios, and implies that output values should reflect the intended purpose of the model, and thus the learnt boundary is essentially correct. The rest of the above definitions are hopefully self-explanatory; for the sake of conciseness, we will only elaborate on our definition of the confidentiality property [56]. Let us consider a classification problem: mapping the items of a data space DS to categories of interest belonging to a set $C=(C_{1},\ldots C_{n})$. A representative sample S⊊DS is used to tabulate a partial classification function f:S→C, obtaining a labeled *training set*, which by abuse of notation we shall also call *f*. We use the training set *f* to *train* an ML model that will be able to compute another function F:DS→C. Finally, we deploy *F* into production, using it to classify individuals from DS as needed. This standard procedure may disclose ML data, for example if the entries in *f* can be inferred from *F*. (For instance, if *F* is computed using the Nearest-Neighbor technique (i.e., $\forall x\in DS,F\left(x\right)=f\left(p_{x}\right)$ where $p_{x}$ is the point in *S* closest to *x* according to some domain distance), *f* is integral part of the definition of *F* and is therefore fully disclosed to the external service whenever *F* is deployed.) Our definition expresses the confidentiality property of a training set asset [57] as follows: a training set *f* is confidential if, observing the execution of a ML model *F* trained on *f*, one is able to infer the same information about any entry e∈f as by observing $F^{\prime }$, obtained using the training set f−{e}+{r}, where *r* is a random entry. The same definition of confidentiality holds for validation and augmented data assets. The resulting threat identification is summarized in Table 8, Table 9 and Table 10, where we also report some known attacks exploiting vulnerabilities to STRIDE-AI threats.

### 5.2. Threats Prioritization

Threat-ranking techniques are used to associate a security risk level to each threat. A popular technique is represented by the so-called *bug bars*, which come in the form of tables listing the criteria used to classify bugs. Recently, Microsoft has released a bug bar [25] to rank ML threats, focusing on intentional malicious behavior against ML-based systems. However, threat prioritisation bug bars are not always easy to explain to users without security expertise. One of the first developed methods to assess the severity of threats is DREAD, an acronym referring to five categories (*Damage Potential*, *Reproducibility*, *Exploitability*, *Affected Users* and *Discoverability*). DREAD, which was designed to complement STRIDE, assigns to each threat a value from 1 to 10. As it turned out that it can lead to inconsistent results due to the intrinsic subjectivity of the rating process [69], the DREAD scaled rating system is no longer recommended for exclusive use; yet, it is still used for quick preliminary threat assessments, as we will do for the case study described in the next section.

## 6. Use Case

We will now apply STRIDE-AI to a real-world use case selected from the AI-ML applications developed in *TOREADOR* (https://cordis.europa.eu/project/id/688797 (accessed on 26 July 2022)) H2020 project. The cyber-security of such applications is a goal of another H2020 project: *THREAT-ARREST* (https://cordis.europa.eu/project/id/786890 (accessed on 26 July 2022)). Our focus is on a scenario contributed by the Light-source company (henceforth LIGHT), one of the major European renewable energy providers, who is a partner in both the above projects. Specifically, the energy pilot use case we consider is a system aimed at maximizing the efficiency of solar energy, where AI-ML technologies are employed for hardware failure prediction (avoiding power cuts) and power flow optimization (avoiding waste and minimizing costs) on the energy grid.

### 6.1. Architecture Specification

**Figure 3:**
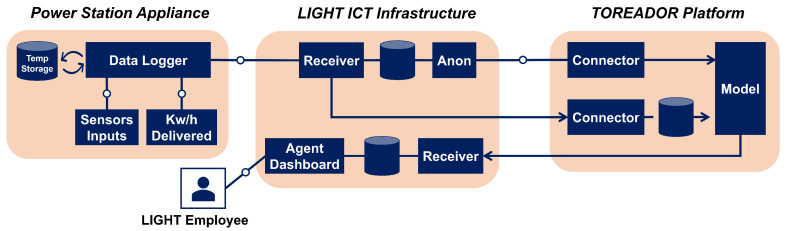
The LIGHT-TOREADOR predictive maintenance architecture.

In the following, we provide a brief description of the LIGHT’s architecture together with its interface with the TOREADOR one. The use case deals with a predictive maintenance scenario in a large-scale asset example. Figure 3 shows an abstraction of the LIGHT-TOREADOR predictive maintenance architecture, and its operation is described by the steps below:

1. Sensors placed at power station sites send data about power and other variables to a local Data Logger card equipped for network communication. The Data Logger has a local root-of-trust.
2. The Data Logger card forwards the data to a Receiver within the LIGHT ICT infrastructure.
3. The Anonymity (Anon) agent retrieves the data and applies some pre-processing, including metadata removal. Removal is not performed for training data, whose metadata are dummy.
4. Pre-processed input data is streamed to a Connector on the TOREADOR platform.
5. Input data is stored in the TOREADOR platform, then fed to an ML model for training/validation or failure prediction.
6. In production, inference (Raw Prediction Data) is returned to the LIGHT platform (predictions are not returned during training).
7. LIGHT employees log into a Dashboard on the LIGHT platform and drill into the inferences.
8. Raw Prediction data is used for further action (including pro-actively maintaining components at the power station).

LIGHT’s monitoring system, whose goal is to provide information to its user on the operation of the solar farms in a timely and concise manner, collects two main categories of measurements (primary measurements and derived ones) through several equipment devices’ monitoring: G59 relay, inverters, and PV string combiners. For the purposes of this use case, we assume only the G59 monitoring device as significant, which has data logging capabilities and is able to keep a registry list with the event codes. The monitor delivers the following categories of data:

- Active energy
- Reactive energy
- Active Power
- Reactive power
- Voltage levels
- Current levels
- Frequency levels
- Power factor
- G59/Alarms

The monitoring data are summarized in a production table which records hourly snapshots of the plant activity. The attributes in this table are:

- report date and time—Store the snapshot date and time.
- production unit id—References the units used to measure the electrical power produced (the unit used to measure energy production are usually KWh (kilowatt hours) or MWh (megawatt hours)).
- quantity—The total energy produced during the report date until the report time.

### 6.2. Metadata

Besides the monitor logs and production table, the plant’s data assets include metadata that describe the facility. They include:

- power plant code—A unique internal code used to designate each plant.
- description—Information related to that power plant, stored in unstructured format.
- location—The power plant’s location, stored as GPS coordinates.
- active—If the power plant is currently active or not.
- date active from—The date when the power plant became active.
- date active to—The date when the power plant stopped being active.

Additional metadata are related to the organization (or the individual) owning and running the plant.

### 6.3. Applying STRIDE-AI

**Figure 4:**
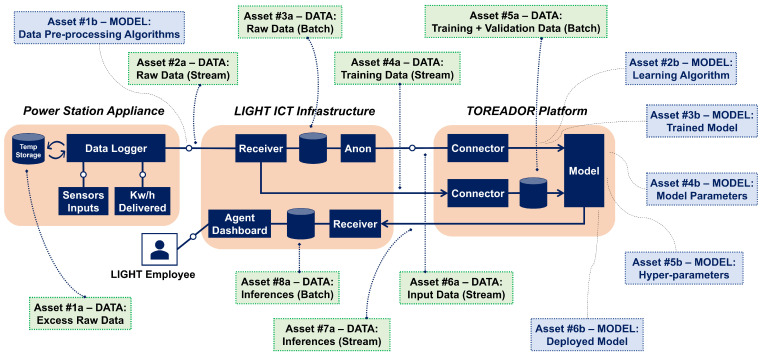
The identified data and model assets.

With reference to the TM process in Table 4, a security expert applying the STRIDE-AI methodology to the power plant has a ready-made set of objectives (the $CIA^{3}-R$ hexagon) and assets categories (the ones on Figure 2). The expert starts by performing architecture decomposition and identifying the relevant assets. For example, the annotations provided in Figure 4 correspond to the data and model assets identified within the predictive maintenance architecture.

**Figure 5:**
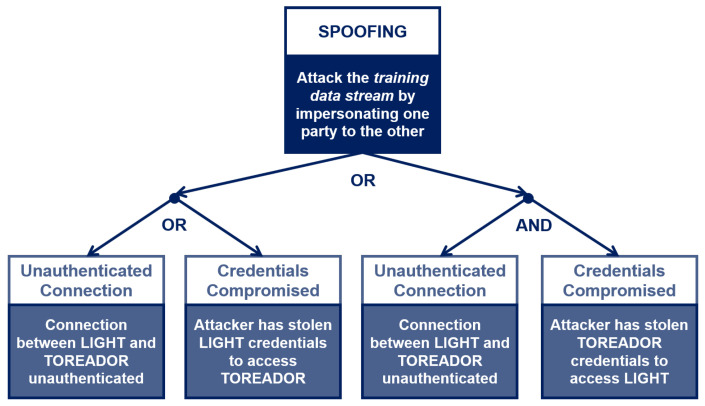
The Spoofing threat tree for the Training Data Stream asset [3].

**Figure 6:**
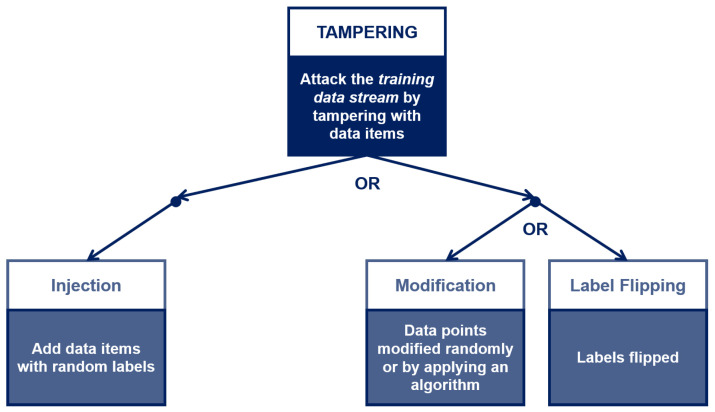
The Tampering threat tree for the Training Data Stream asset [3].

For each asset, the expert chooses a set of properties of interest within $CIA^{3}-R$. To limit the scope of our discussion, let us consider only two assets—*training data stream* (Asset #4a) and *trained model* (Asset #3b) – belonging to the macro-category Data and Model, respectively (The complete mapping of all identified assets, properties and threats is provided in the Appendix A (Table A1). The training data stream asset belongs to the *Labeled Data* asset category; therefore, it should exhibit the Integrity and Authenticity properties to prevent known failures (see Table 8). (Other asset categories will have different property profiles. For example, the library used for training the ML model will need Integrity and Authenticity (to ensure its code has not been tampered with) and Availability (to make sure it will be capable of performing the training or inference task within the deadline).) With regard to the trained model asset, it should exhibit the Integrity and Availability properties (see Table 9). The third step is *threat identification*. Again from Table 8 and Table 9, the security expert identifies (*Tampering* and *Spoofing*) and (*Tampering* and *DoS*) as the threats corresponding to the data and model asset’s property profile, respectively. The next step of the TM process is *vulnerabilities identification*, where the security expert interacts with the system developers to describe under which conditions the threats associated to the assets can materialize. For asset #4a, the *Tampering* threat corresponds to attackers making changes or injecting spurious data into the sensor data stream, while the *Spoofing* threat corresponds to attackers posing as the TOREADOR ML model to the LIGHT data platform, and as the LIGHT platform to the TOREADOR model. The expert needs to assess whether the threats are *atomic* or *composite*, i.e., they require one or more conditions to hold for being exploited. The (simplified) *threat trees* for the *Spoofing* and *Tampering* threats are shown in Figure 5 and Figure 6, respectively.

The *Spoofing* threat tree corresponds to a standard failure mode of distributed platforms. The *Tampering* threat tree is more ML-specific, as it affects the training data stream by injecting spurious data items (i.e., adding data that do not come from LIGHT, with random or chosen labels) or by modifying data items that come from LIGHT by flipping the labels. The difference between the *Tampering* threat sub-trees (Figure 6) is relevant for the expert’s assessment, as the leftmost sub-tree can be deleted as the expert knows that the data items are signed by the Data Logger cards, but not the rightmost one: Loggers’ signature would not prevent label flipping on training data, as labels are added on the LIGHT platform before streaming the training set to TOREADOR.

**Figure 7:**
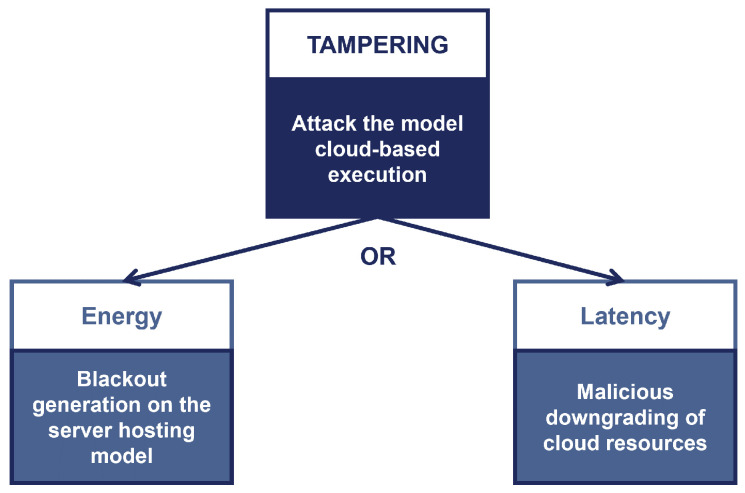
The Tampering threat tree for the Trained Model asset.

As for asset #3b, the *Tampering* threat corresponds to attackers capable of interfering with the model remote execution. As the threat tree in Figure 7 shows, the attacker can mount energy-latency attacks [70]. For instance, someone with remote platform access might slow down the execution time (e.g., by excluding the GPU and switching from GPU to CPU execution, resulting in a doubling of latency). The latency that is generated causes inference values to be out of date.

Finally, we compute the threat priorities by utilizing a DREAD scorecard, whose ratings are shown in Table 11. For the training data stream asset, the potential damage that could result from a spoofing attack includes backdoor generation and substitution of data about power emission or consumption, which could alter metrics and reporting. The expertise required to perform spoofing varies depending on which branch of the *Spoofing* threat tree is considered. As the TOREADOR platform supports a two-factor authentication, in case the LIGHT users’ credentials consist only of a username and password, it may prove easier to gain access to TOREADOR by password cracking or by stealing the credentials (e.g., via phishing emails). An attack exploiting the *Spoofing* threat may result in tampering. If an attacker is able to tamper with the training data stream, she can perform label flipping or other types of data manipulation. The exploitation of this threat depends on the presence of constraints on data manipulation; as labels are added on the LIGHT platform, while data points are collected and signed by Data Loggers, adding new data with random labels requires more effort than flipping labels of existing data to obtain a particular output in the presence of certain input values.

## 7. Discussion

The primary objective of the proposed threat methodology is to reduce the gap between security practitioners and AI experts via a structured approach to identifying, quantifying, and addressing threats to ML. Following STRIDE-AI, an AI-ML-based system can be decomposed into relevant components, and each individual component is analyzed for its susceptibility to threats. Once the failure modes have been identified, they need to be mapped to the threats and the properties such threats may endanger. A benefit of our asset-centered approach is that any threat analyst can easily check which properties are violated as a result of a determined failure, thereby capturing the corresponding security risks of the asset under consideration. By contrast, the approach taken by most of the threat analysis methodologies available in the literature focuses on documenting adversarial tactics and techniques based on real-world observations and providing a static taxonomy of adversarial actions [29,30,31]. One of the sources of difficulty in ML security is the fact that vulnerabilities cannot be comprehensively identified. To be truly effective, the threat analysis should not be accomplished only once because fully enumerating all the failure phenomena of the entire ML-based system at the first attempt, taking into account all possible input-output data, is an infeasible task. As a result, failure modes need to be continuously updated based on additional information about the system itself and the latest research trends on weaknesses and vulnerabilities. In a sense, this corresponds to a *collaborative metadata* approach. Moreover, in order to evaluate the security risks in each failure mode, it is also essential to consider the system’s purpose, its operating environment, and assumption about attackers’ ability. This allows for comprehensive prioritization, also according to factors such as severity, frequency, and detectability.

## 8. Conclusions and Future Work

Currently, there is no systematic process or well-established technique for identifying the vulnerabilities and threats targeting ML components throughout the entire AI pipeline. This research work builds on the urgent necessity to provide any security practitioner with a customized threat modeling methodology for the specific AI pipeline and related ML models. We propose STRIDE-AI as an effective and practical property-driven methodology for assessing the security of AI-ML-based systems. The application of the STRIDE-based threat analysis to the AI-ML domain is relatively simple and has its foundation in the proper identification of critical elements (AI assets) within the ML life-cycle. Such a methodology has the potential to assist AI cybersecurity experts, ML developers, and data scientists in collaboratively identifying ML threats to devise secure ML solutions. However, we are well aware that no threat identification method is effective without providing guidance in selecting the security controls needed to mitigate the identified threats. Unfortunately, no security control framework specifically designed for ML models is currently available. Conventional security controls need to be complemented by ML-oriented security controls and mapped to the core functionalities of ML-based systems they protect and to the weaknesses that threats exploit in these systems. Following [33], future research may explore how available controls in widely used standards, such as ISO 27001 and NIST Cybersecurity framework, can effectively alleviate the harm of identified threats, but also how to carefully choose controls designed for and applicable only to the ML setting. Addressing this issue may also involve the joint use of diverse technologies. For instance, one promising approach might be to employ a *trust-enabling* environment for ML models training and operation, as discussed in [71]. We argue that *Distributed Ledger Technologies* (DLTs) can provide a complete security control framework for ML [72], as DLT usage for $CIA^{3}-R$ properties may render interference with ML inference results less attractive for attackers.

## Figures and Tables

**Table 1:** ML data assets and the corresponding answers to FMEA asset and failure modes questions.

ML Data Asset	Answers to FMEA Asset Questions	Answers to FMEA Failure Modes Questions
*Functional requirements* model the domain of interest, the problem to be solved, and the task to be performed by the ML model. *Non-functional requirements* identify architectural (hardware) and code (software) needs.	- Requirements support a clear understanding of the ML model’s business context and goals. - Requirements must provide a definition of the business goals to be achieved by the ML system, along with the data required to achieve them. - Requirements must not identify a specific model type to be used in the AI system.	- Unclear requirements may lead to inaccurate AI-ML models. - Requirements may not take into account the adverse effect of non-functional properties mandated by regulations. - Requirements may underestimate the severity of information leaks.
*Raw Data* refers to any type of information gathered at the Data Management stage, before it is transformed or analyzed in any way.	- Raw data constitute the primary source of information needed to achieve the model’s business goals. - Raw data provide an initial data pool (not ready for analysis) for use in the subsequent stages of the AI life-cycle.	- Raw data may not be sufficiently representative of the domain or unfit the AI-ML model business goal (e.g., due to sample size and population characteristics). - Raw data volume does not always imply representativeness: if data selection is biased towards elements that have similar characteristics (*selection bias*) even a large set of raw data will not be representative enough.
*Pre-processed data* refers to raw data transformed (cleaned, organized) to feed an AI-ML model.	- Pre-processed data create a data set suitable for ML-based analysis.	- Pre-processed data may cause incorrect estimates of the ML model’s performance if data preparation is applied before splitting the data for model evaluation. - Performing pre-processing on the entire data set may result in *data leakage*, where the ML model is unknowingly exposed to information about test or validation data as part of the training set.
*Labeled Data* refers to sets of multi-dimensional data items used at the Model Learning stage. This data is tagged with informative labels, for the purpose of training supervised ML models.	- Labels make data useful in supervised ML setups. - ML algorithms may use initial labeled data to work with additional unlabeled data.	- Labeled data fail when enough items are deleted or omitted, a sufficient number of spurious labelled data is included into the data set, or enough labels are flipped. - Labeled data may be tampered with to deviate a classifier from its expected behavior.
*Validation Data* is also used at the model learning stage, but differs from ordinary labeled data in usage and, usually, in the circumstances of its collection. Validation data sets are mostly used to perform an evaluation of the ML model in-training, e.g., by stopping training (early stopping) when the error on the validation set increases too much, as this is considered a sign of over-fitting.	- Validation data provides an unbiased evaluation of a model’s fitness on the training data set, while tuning the hyper-parameters. - Validation data helps to deal with over-fitting.	- Validation data may fail when labelled data items are manipulated. - If tampered with, validation data items can affect how the error computed on the validation data set fluctuates during training. - Even a single modification on the validation set may be enough for introducing a spurious error increase that could cut the training short.
*Augmented Data* is labeled data that is complemented at the model tuning stage by additional data produced by transformations or by generative ML models. Augmentation increases labeled data sets’ diversity to prevent over-fitting.	- Augmented data helps to solve the problem of data deficiency by increasing the amount of data available in the training data set. - Data augmentation can be performed in data-space or feature-space. - Augmented data are supposed to prevent over-fitting.	- Augmented data sets may fail due to inconsistency with the training set they are derived from. - Heuristic data augmentation schemes are often tuned manually by humans, and defective augmentation policies may cause ML models to lose rather than gain accuracy from the augmented data.
*Held-out Test Cases* (HTCs) are inputs used to test ML models in production, i.e., in the Model Maintenance stage. HTCs include special inputs of high interest for the application.	- The rationale for HTCs is that even if an ML model keeps showing good accuracy, its performance on specific inputs may become unacceptable.	- HTCs fail when the ML model’s accuracy metrics computed on them does not correspond to the business goals of the application. - Careless selection of HTCs can trigger unneeded model retraining.
*Inferences* are results computed by ML models based on real inputs, according to the task of interest in the Model Deployment and Model Maintenance stages.	- Inferences serve to produce actionable outputs when live data run into ML models.	- Inferences may fail by showing high entropy, i.e., conveying little information useful for the ML task at hand.

**Table 2:** ML model assets and possible answers to FMEA asset and failure modes questions.

ML Model Asset	Answers to FMEA Asset Questions	Answers to FMEA Failure Modes Questions
*Data pre-processing algorithms* are techniques employed to improve information quality by cleaning, integrating and transforming data.	- Data pre-processing has the purpose to convert incomplete or defective raw data into improved training data to provide improved AI-ML model’s performance. - Data pre-processing algorithms should not degrade raw data value. - Data pre-processing should not harvest information about the data.	- Data pre-processing algorithms fail when errors in the definition of the data conversion generate flawed or defective training data. - Data pre-processing may be used to achieve other data properties (e.g., anonymity).
*Hyper-parameters* (HPs) are parameters associated with a AI-ML model that do not depend on the input data, and whose value is set before the learning process begins. They are defined by trial-and-error, using model space search techniques.	- HPs are used for model-space search and identification of the best AI-ML model. - Their value defines the training and structure of the AI-ML model. - HPs should not change during training.	- HPs may fail to deliver the size and configuration for the model that makes its training and operation feasible. - HPs may be used as fitting knobs, e.g., tampering with them to make the model over-fitted to specific training data.
*Learning algorithms* are procedures for adjusting the parameters of ML models, and come in two flavors: *offline* algorithms do not continue to train after deployment (the trained system is frozen), while *online* learning algorithms operate in a continuous learning mode after deployment.	- Learning algorithms should estimate a target function that best maps input variables to an output variable. - Learning algorithms should not impair data representational integrity.	- An improper choice of the learning algorithm may adversely affect the desired accuracy. - Online settings may increase vulnerability exposure because attackers have more chances to drift the AI system from the intended operational use case.
*Model parameters* are variables that are internal to the model (e.g., the weights in a NN, or the centroids in a clustering algorithms) and whose value can be computed by fitting the given input data to the model.	- Parameters determine the inferences (i.e., the actual outputs) computed by the AI-ML model. - Parameters should not reveal information about the training data or the HPs.	- Parameters fail when the AI-ML model’s output computed according to them is such that the model’s performance in executing the task (classification, prediction, anomaly detection) is poor. - Parameters can be used as covert channels to encode hidden information in the inferences.
*Trained models* are AI-ML super-visioned models whose internal parameters have been adjusted by training to achieve a minimum of the error function that defines the distance between actual and expected outputs.	- Trained models perform a ML task (like regression, prediction or anomaly detection) on test input data.	- Trained models fail when the discrepancy between the training data seen during the learning process and inference is so high to cause a drop in performance in production.
*Deployed models* are AI-ML supervisioned models whose parameters are assumed to be stable, and to which users can submit inputs and receive inference outputs.	- Deployed models perform a task (like regression, prediction or anomaly detection) computing inferences in production.	- Deployed models fail when the AI-ML task is not performed at the desired performance (e.g., accuracy) level. - Deployed models can be doctored to compute inferences aimed to benefit malicious third parties.

**Table 3:** ML artefact assets and possible answers to FMEA asset and failure modes questions.

ML Artefact Asset	Answers to FMEA Asset Questions	Answers to FMEA Failure Modes Questions
The *ML model architecture* provides instances of the ML life-cycle components and combines them to a specific multi-stage AI pipeline.	- The model architecture provides guidance toward the software/hardware implementation of the AI pipeline. - The architecture must not allow inferring details of the training or inference algorithms used in the AI pipeline.	- The architecture may be too vague or incomplete to guide pipeline implementation. - The architecture may disclose details on the interfaces between the pipeline stages. - The architecture may disclose details of the training or inference algorithm, facilitating design of vandalism or attacks.
The ML model’s *hardware design* translates the ML models parameters and hyper-parameters into a microprogram, FPGA or neuromorphic circuit design.	- The hardware design includes all design and optimization choices for building the hardware implementation of a ML model. - The hardware design must not allow inferring details of the training or inference algorithms by physical inspection.	- The hardware design choices may support physical side-channels disclosing the model parameters as well as the details of the training or inference algorithm.
*Data and metadata schemata* are definitions of the semantics of the data artefacts fed to (or generated by) specific ML applications. They may be built by specializing a top-level standard concept-base (like ML-Schema, proposed by the W3C Machine Learning Schema Community Group).	- Data and metadata schemata support the interpretability and interoperability of data among ML models as well as back-to-back, seamless connection between the stages of a AI pipeline. - Data and metadata schemata must not expose the nature and purpose of the ML models used in the application.	- Metadata may not deliver human interpretability or interoperability of ML models result. - Metadata may facilitate the extraction of the original training set data from the model.
*Learned data indexes* are ML models to map a key to the position of a data point within a sorted or unsorted array.	- A learned index can learn the sort order or structure of lookup data points and use this information to predict the position or existence of records. - The learned index should not disclose information on the data distribution.	- Learned indexes can disclose information to the data distribution. - Learned indexes can degrade to low performance.

**Table 4:** A 5-step TM process.

Step		Description
1	Objectives Identification	*States the security properties the system should have.*
2	Survey	*Determines the system’s assets, their interconnections and connections to outside systems.*
3	Decomposition	*Selects the assets that are relevant for the security analysis.*
4	Threat Identification	*Enumerates threats to the system’s components and assets that may cause it to fail to achieve the security objectives.*
5	Vulnerabilities Identifications	*Examines identified threats and determines if known attacks show that the overall system is vulnerable to them.*

**Table 5:** STRIDE threats in a nutshell.

Threat	Description
Spoofing Identity	*A user takes on the identity of another. For example, an attacker takes on the identity of a system administrator.*
Tampering with Data	*Information in the system is modified by an attacker. For example, an attacker changes a data item.*
Repudiation	*Information about a transaction is deleted in order to deny it ever took place. For example, an attacker deletes a login transaction to deny he ever accessed an asset.*
Information Disclosure	*Sensitive information is stolen and sold for profit. For example, information on user behavior is stolen and sold to a competitor.*
Denial of Service (DoS)	*This involves exhausting resources required to offer services. For example, in a DoS against a data flow the attacker consumes network resources.*
Elevation of Privilege (EoP)	*This is a threat similar to spoofing, but instead of taking on the ID of another, the attacker elevates his own security level to an administrator.*

**Table 6:** Threats vs. $CIA^{3}-R$ properties in STRIDE-AI.

Property	Threat
Authenticity	Spoofing
Integrity	Tampering
Non-repudiability	Repudiation
Confidentiality	Information Disclosure
Availability	Denial-of-Service (DoS)
Authorization	Elevation-of-Privilege (EoP)

**Table 7:** ML-specific $CIA^{3}-R$ hexagon.

Property	ML-Specific Definition
Authenticity	*The output value delivered by a model has been verifiably generated by it.*
Integrity	*Information used or generated throughout a model’s life-cycle cannot be changed or added to by unauthorized third parties.*
Non-repudiation	*There is no way to deny that a model’s output has been generated by it.*
Confidentiality	*Using a model to perform an inference exposes no information but the model’s input and output.*
Availability	*When presented with inputs, the model computes useful outputs, clearly distinguishable from random noise.*
Authorization	*Only authorized parties can present inputs to the model and receive the corresponding outputs.*

**Table 8:** Mapping data assets’ failure modes to $CIA^{3}-R$ hexagon.

Data Asset	Properties	Threats	Known Attacks
Requirements	Availability	DoS	*While no direct attacks to requirements have been reported, unexpected legal liabilities deriving from defective requirements have been described in a number of cases [58], including ML models for medical diagnostics.*
Raw Data	Authenticity, Confidentiality, Availability, Authorization	Spoofing, Disclosure, DoS, EoP	*Attacks by data owners introduce selection bias on purpose when publishing raw data in order to affect inference to be drawn on the data. Reported examples [59] include companies who release biased raw data with the hope competitors would use it to train ML models, causing competitors to diminish the quality of their own products and consumer confidence in them. In perturbation-style attacks, the attacker stealthily modifies raw data to get a desired response from a production-deployed model [35]. This compromises the model’s classification accuracy.*
Pre-processed data	Integrity, Availability	Tampering, DoS	*Attacks that occur in the pre-processing stage (especially in the context of applications processing images) may mislead all subsequent steps in an AI-ML life-cycle. As an example, image-scaling attacks allow attackers to manipulate images so that their appearance changes when scaled to a specific size.*
Labeled Data	Authenticity, Integrity	Spoofing, Tampering	*Append attacks target availability by adding random samples to the training set to the point of preventing any model trained on that data set from computing any meaningful inference. Other modifications to the training data set (backdoor or insert attacks) jeopardize the ML model’s integrity by trying to introduce spurious inferences [60]. Attackers randomly draw new labels for a part of the training pool to add an invisible watermark that can later be used to “backdoor” into the model.*
Augmented Data	Integrity, Availability	Tampering, DoS	*Adversarial data items tailored to compromise ML model inference can be inserted during data augmentation [61] in order to make them difficult to detect.*
Validation Data	Integrity, Availability	Tampering, DoS	*Attacks can shorten the training of the ML model by compromising just a small fraction of the validation data set. “Adversarial” training data generated by these attacks are quite different from genuine training set data [62].*
Held-Out Test Cases	Integrity, Confidentiality, Availability	Tampering, Disclosure, DoS	*Evaluating an ML model’s performance on HTCs involves reducing all of the information contained in the HTCs outputs to a single number expressing accuracy. The literature reports* slicing attacks *[63], which poison the held-out data set to produce misleading results. Slicing attacks introduce specific slices of data that doctor the model’s accuracy, making it very different from how it performs on the in-production data set.*
Inferences	Authenticity, Integrity, Availability, Authorization	Spoofing, Tampering, DoS, EoP	*Inferences need to carry informative content. The literature reports “eavesdropping attacks” (a survey can be found in [64]) to distributed ML models involving eavesdropping on inferences.*

**Table 9:** Mapping model assets’ failure modes to $CIA^{3}-R$ hexagon.

Model Asset	Properties	Threats	Known Attacks
Data pre-processing algorithms	Integrity, Availability	Tampering, DoS	*Flawed schemata negatively impact on the quality of the ingested information used by applications. An adversary can both compromise the program that pre-process data and mount a schema-based denial of service attack which causes the information necessary for pre-processing to be missing.*
Hyper-parameters	Confidentiality, Availability, Authorization	Disclosure, DoS, EoP	*Nefarious abuse of optimization algorithms by adversaries may lead to erroneous tuning of ML models. Due to their influence over ML models’ predictive capabilities (and, in turn, their commercial value), hyper-parameters are subject to stealing attacks. The literature [65] reports* hyper-parameters stealing *attacks that target hyper-parameters used to balance between the loss function and the regularization terms in an objective function.*
Learning algorithms	Integrity, Confidentiality, Availability	Tampering, Disclosure, DoS	*Several deliberate attack techniques, including adversarial examples, aim to subvert ML learning algorithms. Algorithmic leakage due to poor choice of learning algorithms [66] is known to cause extraction of sensitive information.*
Model parameters	Confidentiality, Availability, Authorization	Disclosure, DoS, EoP	*In a model extraction attack [67], an attacker can extract model parameters via querying the ML model. This way, the attacker can build a near-equivalent shadow model that has the same fidelity as the original one.*
Trained models	Integrity, Availability	Tampering, DoS	*In a model poisoning attack [68], an attacker can replace a functional and legitimate model file with a poisoned one. This type of attack is more likely to occur in cloud-based ML models by exploiting potential weaknesses of cloud providers.*
Deployed models	Authenticity, Integrity, Non-repudiation, Confidentiality, Availability, Authorization	Spoofing, Tampering, Repudiation, Disclosure, DoS, EoP	*A wide variety of attacks at inference time try to compromise ML deployed models. These include model inversion, model evasion, membership inference, model reuse and exploration attacks, among others.*

**Table 10:** Mapping artefact assets’ failure modes to $CIA^{3}-R$ hexagon.

Artefact Asset	Properties	Threats	Known Attacks
Model architecture	Authenticity, Confidentiality	Spoofing, Disclosure	*Man-in-the-Middle attacks use knowledge of the pipeline structure and interfaces to inject malicious data tailored to maximise damage.*
Model hardware design	Confidentiality	Disclosure	*Side-Channel attacks to ML hardware implementations attacks use physical observation of the hardware operation to estimate parameters of the ML model implemented by the circuit.*
Data and metadata schemata	Confidentiality	Disclosure	*Model inversion attacks exploit knowledge about a model’s input features (e.g., representation interval, types) for carry out extraction of the original training set data from the model.*
Learned data indexes	Integrity	Tampering	*Index poisoning attacks target the learned index’s data probability distributions to imperceptibly degrade the index performance.*

**Table 11:** DREAD rating for the Spoofing and Tampering threats.

Threats	D	R	E	A	D	Average [Rating]
DATA: Spoofing	5	2	4	2	7	4.0 [Medium Risk]
DATA: Tampering	7	2	6	2	5	4.4 [Medium Risk]
MODEL: Tampering	6	4	5	5	6	5.2 [Medium Risk]

## Data Availability

Not applicable.

## Appendix A

**Table A1:** Complete mapping for the assets identified in the use case.

Use Case Asset	Properties	Threats
#1a. Excess Raw Data	Authenticity, Integrity	Spoofing, Tampering
#2a. Raw Data (Stream)	Authenticity, Integrity	Spoofing, Tampering
#3a. Raw Data (Batch)	Authenticity, Integrity, Authorization	Spoofing, Tampering, EoP
#4a. Training Data (Stream)	Authenticity, Integrity	Spoofing, Tampering
#5a. Training + Validation Data (Stream)	Authenticity, Integrity, Non-repudiability, Authorization	Spoofing, Tampering, Repudiation, EoP
#6a. Input Data (Stream)	Authenticity, Integrity, Non-repudiability	Spoofing, Tampering, Repudiation
#7a. Inferences (Stream)	Authenticity, Integrity, Non-repudiability, Availability	Spoofing, Tampering, Repudiation, DoS
#8a. Inferences (Batch)	Authenticity, Integrity, Availability, Authorization	Spoofing, Tampering, Dos, EoP
